# Supplementary material for: Characteristics of 5015 Salivary Gland Neoplasms Registered in the Hiroshima Tumor Tissue Registry over a Period of 39 Years
Source: J Clin Med. 2019 Apr 26;8(5):566. doi: 10.3390/jcm8050566 (PMC6571736; doi:10.3390/jcm8050566)
Supplement: Supplementary file 1 [file jcm-08-00566-s001.pdf]

Supplementary Tables

Table S1. Histological frequencies and locations in 692 malignant major salivary gland tumors.

	Histological types (WHO)	Parotid		Submandibular		Sublingual		NOS		Total
		M	F	M	F	M	F	M	F	
1	Mucoepidermoid carcinoma	68	57	18	11	1	1	1	0	157
2	Adenoid cystic carcinoma	27	49	21	37	7	8	0	1	150
3	Carcinoma ex pleomorphic adenoma	47	23	14	12	4	0	0	0	100
4	Malignant lymphomas	15	27	6	9	0	1	2	1	61
	Malignant lymphoma, non-Hodgkin, NOS	(3)	(9)	(1)	(4)	(0)	(0)	(1)	(1)	(19)
	Marginal zone B-cell lymphoma, NOS	(5)	(9)	(2)	(0)	(0)	(1)	(0)	(0)	(17)
	Malignant lymphoma, large B-cell, diffuse, NOS	(2)	(6)	(0)	(2)	(0)	(0)	(0)	(0)	(10)
	Follicular lymphoma, NOS	(3)	(2)	(1)	(2)	(0)	(0)	(1)	(0)	(9)
	Burkitt lymphoma, NOS	(0)	(1)	(2)	(0)	(0)	(0)	(0)	(0)	(3)
	Mantle cell lymphoma	(1)	(0)	(0)	(0)	(0)	(0)	(0)	(0)	(1)
	NK/T-cell lymphoma, nasal and nasal-type	(1)	(0)	(0)	(0)	(0)	(0)	(0)	(0)	(1)
	Adult T-cell leukemia/lymphoma (HTLV-1 positive)	(0)	(0)	(0)	(1)	(0)	(0)	(0)	(0)	(1)
5	Adenocarcinoma, NOS	25	15	9	2	0	1	0	0	52
6	Acinic cell carcinoma	18	22	2	2	0	0	0	0	44
7	Poorly differentiated carcinoma	10	6	6	4	0	0	1	0	27
8	Secretory carcinoma	15	5	0	3	0	0	0	0	23
9	Salivary duct carcinoma	16	0	2	0	0	0	0	0	18
10	Epithelial-myoepithelial carcinoma	6	9	1	0	0	0	1	1	18
11	Myoepithelial carcinoma	5	3	2	5	0	0	0	0	15
12	Basal cell adenocarcinoma	5	4	0	1	0	0	0	0	10
13	Squamous cell carcinoma	4	1	1	1	0	0	0	0	7
14	Oncocytic carcinoma	2	0	1	0	0	0	0	0	3
15	Intraductal carcinoma	1	1	0	0	0	0	0	0	2
16	Carcinosarcoma	1	0	0	0	1	0	0	0	2
17	Carcinoma, NOS	0	0	1	0	0	0	0	0	1
18	Lymphoepithelial carcinoma	1	0	0	0	0	0	0	0	1
19	Sebaceous adenocarcinoma	1	0	0	0	0	0	0	0	1
	Totals	267	222	84	87	13	11	5	3	692

Abbreviations: M: male; F: female; NOS: not otherwise specified.

Table S2. Histological frequencies and locations in 325 malignant minor salivary gland tumors.

	Histological types (WHO)	Palatal		Upper lip		Lower lip		Buccal		Lingual	
		M	F	M	F	M	F	M	F	M	F
1	Adenoid cystic carcinoma	24	24	2	3	1	3	2	12	4	12
2	Mucoepidermoid carcinoma	11	23	1	4	1	4	7	7	7	7
3	Myoepithelial carcinoma	7	6	0	1	1	0	0	2	1	0
4	Adenocarcinoma, NOS	3	3	1	1	0	0	1	0	2	1
5	Acinic cell carcinoma	1	2	2	1	0	0	0	6	0	0
6	Carcinoma ex pleomorphic adenoma	2	6	0	0	0	0	0	2	0	0
7	Poorly differentiated carcinoma	2	1	0	0	0	0	0	1	0	0
8	Malignant lymphomas	0	1	0	0	0	1	0	2	0	0
	Marginal zone B-cell lymphoma, NOS	(0)	(1)	(0)	(0)	(0)	(0)	(0)	(2)	(0)	(0)
	Mantle cell lymphoma	(0)	(0)	(0)	(0)	(0)	(1)	(0)	(0)	(0)	(0)
9	Clear cell carcinoma	1	1	0	0	0	0	0	1	0	1
10	Epithelial-myoepithelial carcinoma	1	2	0	0	0	0	0	0	0	0
11	Basal cell adenocarcinoma	1	1	0	0	0	0	0	0	0	0
12	Polymorphous adenocarcinoma	1	1	0	1	0	0	0	0	0	0
13	Carcinoma, NOS	0	0	0	0	1	0	0	0	0	0
14	Oncocytic carcinoma	0	0	0	0	0	0	1	0	0	0
	Totals	54	71	6	11	4	8	11	33	14	21

Abbreviations: M: male; F: female; NOS: not otherwise specified.

Table S2. continued

	Histological types (WHO)	Floor of mouth		Retro-molar		NOS		Total
		M	F	M	F	M	F	
1	Adenoid cystic carcinoma	14	13	2	1	1	3	121
2	Mucoepidermoid carcinoma	13	7	5	6	2	4	109
3	Myoepithelial carcinoma	3	1	0	0	1	0	23
4	Adenocarcinoma, NOS	1	0	3	0	0	0	16
5	Acinic cell carcinoma	0	0	0	1	0	0	13
6	Carcinoma ex pleomorphic adenoma	1	0	0	0	0	0	11
7	Poorly differentiated carcinoma	0	0	3	0	1	0	8
8	Malignant lymphomas	1	1	0	0	0	0	6
	Marginal zone B-cell lymphoma, NOS	(1)	(1)	(0)	(0)	(0)	(0)	(5)
	Mantle cell lymphoma	(0)	(0)	(0)	(0)	(0)	(0)	(1)
9	Clear cell carcinoma	0	1	0	0	0	0	5
10	Epithelial-myoepithelial carcinoma	0	0	0	1	0	0	4
11	Basal cell adenocarcinoma	0	1	0	0	0	0	3
12	Polymorphous adenocarcinoma	0	0	0	0	0	0	3
13	Carcinoma, NOS	0	0	0	0	0	1	2
14	Oncocytic carcinoma	0	0	0	0	0	0	1
	Totals	33	24	13	9	5	8	325

Abbreviations: M: male; F: female; NOS: not otherwise specified.

Table S3. Histological frequencies and locations in 3626 benign major salivary gland tumors.

	Histological types (WHO)	Parotid		Submandibular		Sublingual		NOS		Total
		M	F	M	F	M	F	M	F	
1	Pleomorphic adenoma	673	1112	195	357	4	2	7	16	2366
2	Warthin tumor	892	153	6	2	0	0	0	1	1054
3	Basal cell adenoma	43	85	3	2	0	0	0	0	133
4	Myoepithelioma	8	11	2	3	0	0	0	0	24
5	Adenoma, NOS	7	7	1	0	0	0	0	1	16
6	Cystadenoma	1	11	0	0	2	0	0	0	14
7	Hemangioma, NOS	1	3	1	2	0	0	0	0	7
8	Lipoma, NOS	4	1	0	0	0	0	0	0	5
9	Lymphangioma, NOS	2	1	1	1	0	0	0	0	5
10	Sebaceous adenoma	1	0	0	0	0	0	0	0	1
11	Ductal papilloma	0	0	0	0	0	0	1	0	1
	Totals	1632	1384	209	367	6	2	8	18	3626

Abbreviations: M: male; F: female; NOS: not otherwise specified.

Table S4. Histological frequencies and locations in 372 benign minor salivary gland tumors.

	Histological types (WHO)	Palatal		Upper lip		Lower lip		Buccal		Lingual	
		M	F	M	F	M	F	M	F	M	F
1	Pleomorphic adenoma	93	137	22	31	6	4	9	27	2	2
2	Myoepithelioma	5	6	0	1	0	0	0	0	1	0
3	Cystadenoma	0	0	0	0	1	0	0	1	0	0
4	Basal cell adenoma	0	2	0	0	0	0	0	0	1	0
5	Canalicular adenoma	0	0	0	0	0	0	1	0	0	1
6	Adenoma, NOS	0	1	0	0	0	0	0	0	0	0
7	Ductal papilloma	0	0	1	0	0	0	0	0	0	0
8	Fibroma, NOS	0	0	0	0	0	0	0	1	0	0
	Totals	98	146	23	32	7	4	10	29	4	3

Abbreviations: M: male; F: female; NOS: not otherwise specified.

Table S4. continued

	Histological types (WHO)	Floor of mouth		Retro-molar		NOS		Total
		M	F	M	F	M	F	
1	Pleomorphic adenoma	2	5	1	1	4	0	346
2	Myoepithelioma	0	0	0	0	0	0	13
3	Cystadenoma	0	1	1	1	0	0	5
4	Basal cell adenoma	0	0	0	0	0	0	3
5	Canalicular adenoma	0	0	0	0	0	0	2
6	Adenoma, NOS	0	0	0	0	0	0	1
7	Ductal papilloma	0	0	0	0	0	0	1
8	Fibroma, NOS	0	0	0	0	0	0	1
	Totals	2	6	2	2	4	0	372

Abbreviations: M: male; F: female; NOS: not otherwise specified.

Table S5. Annual registered number of cases and age-adjusted registration rates per year per 100,000 in 5005 salivary gland tumors

	Males				Females				All				All	
	Benign		Malignant		Benign		Malignant		Benign		Malignant		All	
Years	Number	Rate	Number	Rate	Number	Rate	Number	Rate	Number	Rate	Number	Rate	Number	Rate
1973–1977	99	1.7	28	0.5	120	1.8	41	0.7	219	1.8	69	0.6	288	2.3
1978–1982	146	2.4	52	0.9	198	2.9	50	0.7	344	2.6	102	0.8	446	3.4
1983–1987	195	2.9	55	0.8	233	3.2	55	0.7	428	3.0	110	0.8	538	3.8
1988–1992	253	3.5	65	0.9	264	3.4	65	0.8	517	3.4	130	0.8	647	4.2
1993–1997	277	3.6	80	1.0	300	3.6	67	0.7	577	3.6	147	0.8	724	4.4
1998–2002	278	3.3	71	0.8	281	3.3	75	0.7	559	3.3	146	0.8	705	4.1
2003–2007	345	4.0	76	0.7	317	3.7	82	0.8	662	3.8	158	0.8	820	4.6
2008–2011	408	5.7	81	1.1	275	4.0	73	0.8	683	4.8	154	0.9	837	5.8
1973–2011	2001	3.5	508	0.8	1988	3.3	508	0.7	3989	3.3	1016	0.8	5005	4.1

Ten cases with unknown patient age were excluded from the total number of 5010 cases.
